# Keystone Veterinary Medical Association

**Published:** 1898-02

**Authors:** W. L. Rhoads

**Affiliations:** Secretary


Secretary.

### KEYSTONE VETERINARY MEDICAL ASSOCIATION.

THE January meeting was called to order by President Leonard Pearson, Tuesday evening, January 11th, with the following present: Drs. J. Cheston Morris, S. J. J. Harger, George V. Fallows, J. D. Houldsworth, W. Horace Hoskins, Charles Lintz, Leonard Pearson, James B. Rayner, W. L. Rhoads, Thomas B. Rayner, H. A. Christmann, C. J. Marshall, A. N. Lushington; also Messrs. Repp, Spindler, Cunningham, Kirby, and Jones, of the Veterinary Department of the University of Pennsylvania.

After the routine business, Drs. Rhoads and Hoskins offered a resolution making the payment of $5.00 membership cover the dues till the succeeding annual meeting; to be acted on at February meeting.

Dr. S. J. J. Harger now read a very entertaining and instructive paper on the "Use of Arecoline in Colic." Its action is similar to that of eserine and pilocarpine combined; it is rapidly eliminated, and successive hourly doses can be given. It is contraindicated in horses with respiratory trouble or with functional alterations of heart, also in pregnant females. Common horses are more susceptible than well-bred ones. Cattle are very susceptible. Dr. C. J. Marshall said he had used the drug in a number of cases, and found it to act the same in 2-grain doses as 2 grains of eserine and 3 grains of pilocarpine would. Dr. Lintz said in impaction colic you always had a frictional sound in the movements of the joints.

The delivery of milk in glass jars was now discussed. The general consensus of opinion being that if bottled in good condition at the dairy, this was undoubtedly the best method.

The President appointed Drs. C. J Marshall and W. H. Hoskins as a committee to answer an article in a paper which condemns this method of serving.

Dr. Hoskins asked what could be done to ascertain the truth of statement made by milkmen who said in their display-card, "dairy under veterinary, sanitary, and chemical inspection."

The President appointed Drs. C. J. Marshall, J. D. Houldsworth, and Charles Lintz to take this matter in charge.

Meeting adjourned till February 8, 1898. DR. W. L. RHOADS,
Secretary.

